# MicroRNA expression profiles in familial hypertrophic cardiomyopathy with myosin-binding protein C3 (*MYBPC3*) gene mutations

**DOI:** 10.1186/s12872-022-02714-6

**Published:** 2022-06-18

**Authors:** Li-rong Lin, Xue-qun Hu, Li-hong Lu, Jia-zhen Dai, Ning-ning Lin, Re-hua Wang, Zhang-xin Xie, Xue-mei Chen

**Affiliations:** 1 Shengli Clinical Medical College of Fujian Medical University, Fuzhou, 350001 China; 2 Department of Cardiology, Fujian Provincial Hospital, Fuzhou, 350001 China; 3 Department of Neurosurgery, The First Affiliated Hospital of Fujian Medical University, Fuzhou, 350001 China; 4 Department of Emergency, Fujian Provincial Hospital, Fuzhou, 350001 China

**Keywords:** Familial hypertrophic cardiomyopathy, MYBPC3, Gene mutation, MicroRNA-sequencing

## Abstract

Familial hypertrophic cardiomyopathy (FHCM) is an autosomal dominant inherited disease caused by mutations in genes encoding cardiac sarcomere proteins. MicroRNAs (miRNAs) play an important role in the pathogenesis of FHCM. In the present study, we aimed to determine the miRNA profile in FHCM patients with myosin-binding protein C3 (*MYBPC3*) gene mutations. We recruited three FHCM patients and age- and sex-matched controls. The three probands all had hypertrophic obstructive cardiomyopathy with severe myocardial hypertrophy, and two of the three had a history of sudden cardiac death, representing a “malignant” phenotype. We then compared the miRNA expression profiles of three FHCM patients carrying *MYBPC3* gene mutations with those of the normal control group using miRNA sequencing technology. Differentially expressed miRNAs were verified using real-time polymerase chain reaction (qPCR). Target genes and signaling pathways of the identified differentially expressed miRNAs were predicted using bioinformatics analysis. A total of 33 significantly differentially expressed miRNAs were detected in the peripheral blood of the three probands, of which 28 were upregulated, including miR-208b-3p, and 5 were downregulated. Real-time PCR confirmed the upregulated expression of miR-208b-3p in FHCM patients (*P* < 0.05). Bioinformatics analysis showed that miR-208b-3p was mainly enriched in 79 target genes including *UBE2V2*, *MED13*, *YBX1*, *CNKSR2*, *GATA4*, and*SOX5/6, *et al. Gene ontology (GO) analysis of target genes showed that miR-208b was mainly involved in the processes of negative regulation of transcription from RNA polymerase II promoter, and regulation of transcription, DNA templated. Kyoto Encyclopedia of Genes and Genomes (KEGG) analysis revealed that the target genes regulated by miR-208b-3p were mainly involved in the Wnt signaling pathway. These findings suggest that FHCM patients with *MYBPC3* gene mutations have a specific miRNA expression profile, and that miR-208b-3p is significantly upregulated in cardiac hypertrophy. Our results also indicate that miRNA-208b-3p activates the Wnt signaling pathway through its target gene to promote cardiac hypertrophy.

## Introduction

Hypertrophic cardiomyopathy (HCM) is an autosomal dominant inherited disease caused by mutations in genes encoding cardiac sarcomere proteins. Pathogenic gene mutations have been discovered in approximately 60% of adult FHCM patients [1–4]. The pathogenesis of FHCM exhibits significant familial clustering, with offspring having a 50% probability of inheriting the pathogenically mutated gene. To date, more than 1400 pathogenic mutation sites have been identified in more than 25 genes [5]. Common pathogenically mutated genes in Chinese populations include *MYH7*,*MYH6*, *MYBPC3*, *TNNT2*, *TNNI3*,*TPM1*, *ACTC1*, *MYL2*, *MYL3*, and *LAMP2* [6], among which myosin-binding protein C3 (*MYBPC3*)has the highest mutation rate, accounting for 30–50% of all FHCM cases [7–9]. Studies have shown that more than 70% of *MYBPC3* mutations in FHCM result in truncated cMyBP-C proteins lacking the C-terminus region [10]. For example, one *MYBPC3* mutation results in a truncated peptide that encodes only the N-terminal 258 amino acids and25 additional, new amino acids [11]. The clinical manifestations of FHCM range from asymptomatic or relatively mild symptoms to heart failure and sudden cardiac death, with FHCM being one of the main causes of sudden cardiac death in adolescents and athletes [12]. However, the clinical phenotypes of FHCM patients with the same gene mutation can vary, and even among the members of the same family, the clinical manifestations, age of onset, and prognosis of patients can also be very different. These findings suggest that there is no direct relationship between gene mutations and clinical phenotypes. These differences are thought to be attributed to different factors including pathogenic genes, environmental cues, and genetic modifiers [13–15].

MicroRNAs (miRNAs) are endogenous non-coding RNAs comprised of approximately 20–25 nucleotides that have regulatory functions. MiRNAs are often considered to be negative regulators of gene expression. MiRNAs can inhibit translation and/or promote mRNA degradation through base pairing with a complementary sequence within the 3′-untranslated region (3′-UTR) of the target gene mRNA transcript. Approximately 60% of protein-coding genes have been found to be regulated by miRNAs at the post-transcriptional level [16–18]. MiRNA sequence screening analysis of many human and animal models of cardiac hypertrophy showed that the expression of some miRNAs (miR-1, miR-133, miR-9, miR-26b, miR-98, miR-181b, and miR-30e) was downregulated, while the expression of other miRNAs (miR-208, miR-155, miR-199, miR-499, miR-26a, miR-21, miR-23a, and miR-29a) was upregulated. Mechanistically, Ye et al. [19] found that miRNA-20b promotes cardiac hypertrophy by inhibiting mitofusin2-mediated inter-organelle Ca^2+^cross-talk. Studies have also shown that miR-29a and miR-29c can be used to distinguish between hypertrophic non-obstructive, aortic stenosis, and obstructive cardiomyopathies [20]. Montgomery et al. [21] found that miRNA-208a antagonists improve cardiac function and the survival rate in mice with cardiac hypertrophy. Therefore, identifying miRNA expression profiles in FHCM patients may further our understanding of the pathogenesis of FHCM and offer new therapeutic avenues for treatment.

In the present study, we examined the miRNA expression profiles of FHCM patients carrying *MYBPC3* gene mutations using high-throughput miRNA-seq analysis and used bioinformatic analyses to predict target genes and signaling pathways. The findings of this study provide new insights into the mechanisms underlying FHCM pathogenesis and new perspectives on therapeutic targets.

## Materials and methods

### Patients

This study protocol was approved by the Ethics Committee of Fujian Provincial Hospital (IRB number: K2017-09-066) and in accordance with the Declaration of Helsinki). All subjects volunteered to participate in this study and provided written informed consent.

Three probands (1 female, 84 years of age, and 2 males, 23 and 65 years of age, respectively) from Han Chinese hypertrophic cardiomyopathy families who were admitted to Fujian Provincial Hospital between October 2017 and October 2018 were recruited as study participants. The clinical diagnostic criteria of HCM were based on the 2014 European Society of Cardiology Guidelines as follows: HCM is defined by a wall thickness ≥ 15 mm in one or more left ventricular (LV) myocardial segments measured by any imaging technique such as echocardiography, cardiac magnetic resonance (CMR), or computed tomography, which could not be explained solely by loading conditions. The clinical diagnosis of HCM in the first-degree relatives of patients with unequivocal disease (left ventricle hypertrophy (LVH) ≥ 15 mm) was based on the presence of an otherwise unexplained increased LV wall thickness ≥ 13 mm in one or more LV myocardial segments, as measured using any cardiac imaging technique (echocardiography, CMR, or CT). Diagnostic criteria for FHCM were based on clinical manifestations and ultrasound diagnosis. In addition to the proband, two or more of the three generations of direct relatives were diagnosed as having HCM or identified as HCM-related sudden death patients. The probands were diagnosed as HCM by two experienced cardiologists based on clinical manifestations, Doppler echocardiography, and electrocardiogram. The control group consisted of sex- and age-matched healthy subjects without cardiac disease. Patients with the following diseases that may cause cardiac hypertrophy were excluded: history of hypertension for over 10 years, rheumatic disease, aortic stenosis, congenital heart disease and metabolic diseases (such as myocardial amyloidosis, Danon disease, and Pompe disease), cardiac hypertrophy of athletes, and other organic heart diseases.

### Screening and identification of mutation sites in pathogenic genes

Genomic DNA was extracted from peripheral blood (5 ml) collected from patients in the FHCM group. High-throughput micro of common pathogenic genes encoding sarcomere proteins was performed in the Shenzhen Huada Clinical Experimental Center (China) using exon capture technology. The sequenced genes were as follows: beta-myosin heavy chain (*MYH7*), myosin-binding protein C(*MYBPC3*), cardiac troponin I and T (*TNNI3*, *TNNT2*), tropomyosin alpha-1 chain (*TPM1*), myosin light chain 3 (*MYL3*), and cardiac α-actin (*ACTC1*).

### Detection of peripheral blood miRNAs in FHCM patients with gene mutations

#### Collection of experimental specimens

Venous blood samples (5 ml) were drawn from three FHCM patients and three healthy people to BD Vacutainer Venous Blood Collection Tubes containing EDTA, then turn tubes up and down many times to mixed blood and anticoagulant fully. Centrifuge 3000 rpm at 4 °C for 10 min, collected supernatants as plasma. The fresh plasma samples was frozen immediately at − 80 °C until using.

#### Total RNA isolation

Total RNA from plasma samples were isolated using TRIzol LS Reagent (Invitrogen, Carlsbad, CA, USA) according to the manufacturer’s instructions. The concentration and purity of RNA were measured using NanoDropND-1000 (NanoDrop Technologies, USA). The extracted RNA samples were of good quality and high purity, thus meeting the standard requirements for miRNA-seq and RT-qPCR analyses.

#### MiRNA sequencing

Total RNA from six plasma samples was used to construct miRNA sequencing library. The TruSeq Small RNA library Preparation Kit (Illumina, San Diego, CA, USA) was used for library preparation through the following steps: (1) 3′-adaptor ligation; (2) 5′-adaptor ligation; (3) cDNA synthesis; (4) PCR amplification; (5) ~ 135 to 155 bp PCR fragments recovery. The libraries were denatured as single DNA strands, captured on Illumina flow cells (Illumina, San Diego, CA, USA), amplified in situ as clusters and finally sequenced for 51 cycles on Illumina NextSeq 500 sequencer. The raw data was analyzed using routine algorithms (KangChen Biotech, Shanghai, China).

### RT-qPCR validation of differentially expressed miRNAs

Total RNA samples were reverse-transcribed (RT) to generate cDNA using a stem-loop RT primer (Primer Premiere 5.0) with the SuperScript III Reverse Transcriptase kit (Invitrogen) according to the manufacturer’s instructions. The RT-qPCR system consisted of 5 μl 2 × Master Mix, 0.5 µl forward primer (10 µM), 0.5 µl reverse primer (10 µM), 2 µl cDNA, and 2 µl double-distilled water. Hsa-miR-423-5p was used as the internal reference. Samples were analyzed in a 96-well plate using a real-time PCR instrument (ViiA 7 Real-time PCR System, Applied Biosystems Technologies, USA). The RT-qPCR conditions were as follows: 95 °C for 10 min, followed by 40 cycles of 95 °C for 10 s and 60 °C for 60 s. Melting curves were generated to evaluate reaction specificity under the following conditions: 95 °C for 10 s, 60 °C for 60 s, and 95 °C for 15 s. The stem-loop RT primers showed in Table 1 and PCR primer sequences used in this study are shown in Table 2.

**Table 1:** Sequences of Stem-loop reverse transcript primers used in this study

miRNA	Stem-loop RT Primer
hsa-miR-2355-5p	5′GTCGTATCCAGTGCGTGTCGTGGAGTCGGCAATTGCACTGGATACGACTTGTCC3′
hsa-miR-200c-5p	5′GTCGTATCCAGTGCGTGTCGTGGAGTCGGCAATTGCACTGGATACGACCCAAAC3′
hsa-miR-26a-1-3p	5′GTCGTATCCAGTGCGTGTCGTGGAGTCGGCAATTGCACTGGATACGACCGTGCA3′
hsa-miR-146b-5p	5′GTCGTATCCAGTGCGTGTCGTGGAGTCGGCAATTGCACTGGATACGACAGCCTA3′
hsa-let-7g-3p	5′GTCGTATCCAGTGCGTGTCGTGGAGTCGGCAATTGCACTGGATACGACGCAAGGC3′
hsa-miR-103a-3p	5′GTCGTATCCAGTGCGTGTCGTGGAGTCGGCAATTGCACTGGATACGACTCATAGC3′
hsa-miR-122-5p	5′GTCGTATCCAGTGCGTGTCGTGGAGTCGGCAATTGCACTGGATACGACCAAACA3′
hsa-miR-208b-3p	5′GTCGTATCCAGTGCGTGTCGTGGAGTCGGCAATTGCACTGGATACGACACAAAC3′
hsa-miR-377-3p	5′GTCGTATCCAGTGCGTGTCGTGGAGTCGGCAATTGCACTGGATACGACACAAAAG3′
hsa-miR-675-3p	5′GTCGTATCCAGTGCGTGTCGTGGAGTCGGCAATTGCACTGGATACGACTGAGCG3′
hsa-miR-423-5p	5′GTCGTATCCAGTGCGTGTCGTGGAGTCGGCAATTGCACTGGATACGACAAAGTC3′

**Table 2:** Sequences of PCR primers used in this study

Gene	Forward (5′–3′)	Reverse (5′–3′)
hsa-miR-2355-5p	F:5′GGGCAATCCCCAGATACAAT3′	R:5′GTGCGTGTCGTGGAGTCG3′
hsa-miR-200c-5p	F:5′GGAGCGTCTTACCCAGCAGT3′	R:5′GTGCGTGTCGTGGAGTCG3′
hsa-miR-26a-1-3p	F:5′GGGGTCCTATTCTTGGTTACT3′	R:5′CAGTGCGTGTCGTGGAGT3′
hsa-miR-146b-5p	F:5′GGGGTGAGAACTGAATTCCA3′	R:5′GTGCGTGTCGTGGAGTCG3′
hsa-let-7g-3p	F:5′GGGGAATGTACAGGCCACT3′	R:5′CAGTGCGTGTCGTGGAGTC3′
hsa-miR-103a-3p	F:5′GGGGAGCAGCATTGTACAGG3′	R:5′CAGTGCGTGTCGTGGAGT3′
hsa-miR-122-5p	F:5′GGGTGGAGTGTGACAATGG3′	R:5′CAGTGCGTGTCGTGGAGT3′
hsa-miR-208b-3p	F:5′GGGGATAAGACGAACAAAAG3′	R:5′CAGTGCGTGTCGTGGA3′
hsa-miR-377-3p	F:5′GGGAATCACACAAAGGCAAC3′	R:5′GTGCGTGTCGTGGAGTCG3′
hsa-miR-675-3p	F:5′GAAGGCTGTATGCCCTCACC3′	R:5′GTGCGTGTCGTGGAGTCG3′
hsa-miR-423-5p	F:5′TGAGGGGCAGAGAGCGA3′	R:5′GTGCGTGTCGTGGAGTCG3′

### GO and KEGG pathway analysis

Gene ontology (GO) project provides a vocabulary to describe gene functions in any organism (http://geneontology.org/), including three domains: biological process, cellular component and molecular function. Fisher’s exact test was used to determine the significance of enriched terms. The lower the *p* value, the more significant the GO terms (threshold is *p* value < 0.05).

Kyoto Encyclopedia of Genes and Genomes (KEGG) pathway analysis is a functional analysis mapping gene to KEGG pathways [22]. The Fisher’s exact test *p* value denotes the significance of pathway terms (the *p* value cutoff is 0.05).

### Statistical analysis

Differential expression of miRNAs was evaluated using the *p* value and fold change (FC) value determined using the paired *t*-test, with differentially expressed miRNAs defined as those with a FC in expression (upregulation or downregulation) greater than 1.5 and a *p* < 0.05. TargetScan (http://www.targetscan.org/) and miRDB (http://mirdb.org/miRDB/), which are currently the mainstream databases for predicting target genes of miRNAs, were selected to jointly predict target genes of differentially expressed miRNAs (TargetScan cutoff parameter is total contex++ score < − 0.05, meanwhile miRdb score > 50). Gene Ontology (GO) and Kyoto Encyclopedia of Genes and Genomes (KEGG) pathway analyses were performed to identify the function of target genes. Fisher’s exact test was used to determine the significance of enriched terms (the *p* value cutoff is 0.05). qPCR results were calculated using the 2^−ΔΔCT^ method.

## Results

### Genetic screening of mutated genes in FHCM patients

**Fig. 1:**
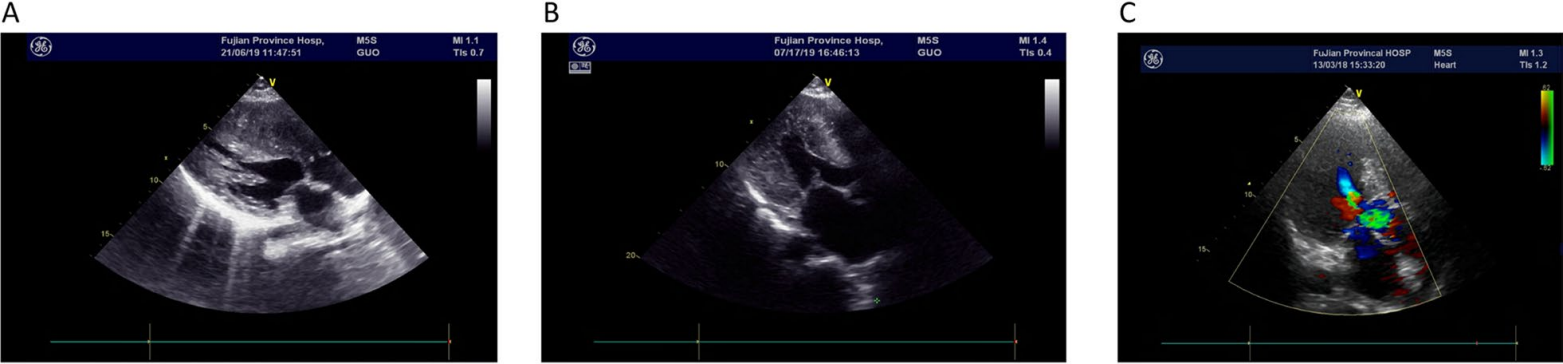
Echocardiography of FHCM patients showed abnormal ventricular septal thickness. **a** Patient 1, female, 27 years old. Echocardiography showed that the left ventricular myocardium showed asymmetric hypertrophy, and the thickest left ventricular wall was 2.38 cm. **b** Patient 2, male, 68 years old. Echocardiography showed that the left ventricular myocardium showed asymmetric hypertrophy, and the thickest left ventricular wall was 2.18 cm. **c** Patient 3, female, 84 years old. Echocardiography showed that the left ventricular myocardium showed asymmetric hypertrophy, and the thickest left ventricular wall was 3.36 cm

High-throughput gene screening was conducted on a total of 16 family members from three FHCM families. Three of the five families had *MYBPC3* gene mutations. The clinical data of FHCM probands with *MYBPC3* gene mutations are shown in Table 3. Figure 1 showed the echocardiography of FHCM patients, whose left ventricular septal thickness more than 15 mm.

**Table 3:** Clinical data of FHCM probands with the *MYBPC3* gene mutations

Family	Sex	Age	Obstructive	Arrhythmia	NYHA classification	Syncope	Family history of SCD	Maximum wall thickness (mm)	Mutation site	Troponin I* (ng/ml)
Case1	Male	65	Yes	No	II	Yes	Yes	30.9	c.3369_3370insC	1.29
Case2	Male	23	Yes	No	I	No	Yes	40.5	c.3624delC	0.20
Case3	Female	84	Yes	Atrial fibrillation	IIII	No	No	33.6	c.3624delC	0.31
Ctrl1	Male	61	No	No	I	No	No	9.8	–	< 0.01
Ctrl2	Male	23	No	No	I	No	No	9.5	–	< 0.01
Ctrl3	Female	78	No	No	I	No	No	10.5	–	< 0.01

The normal value of Troponin I is < 0.1 ng/ml

### MiRNA sequencing preliminary screening

**Fig. 2:**
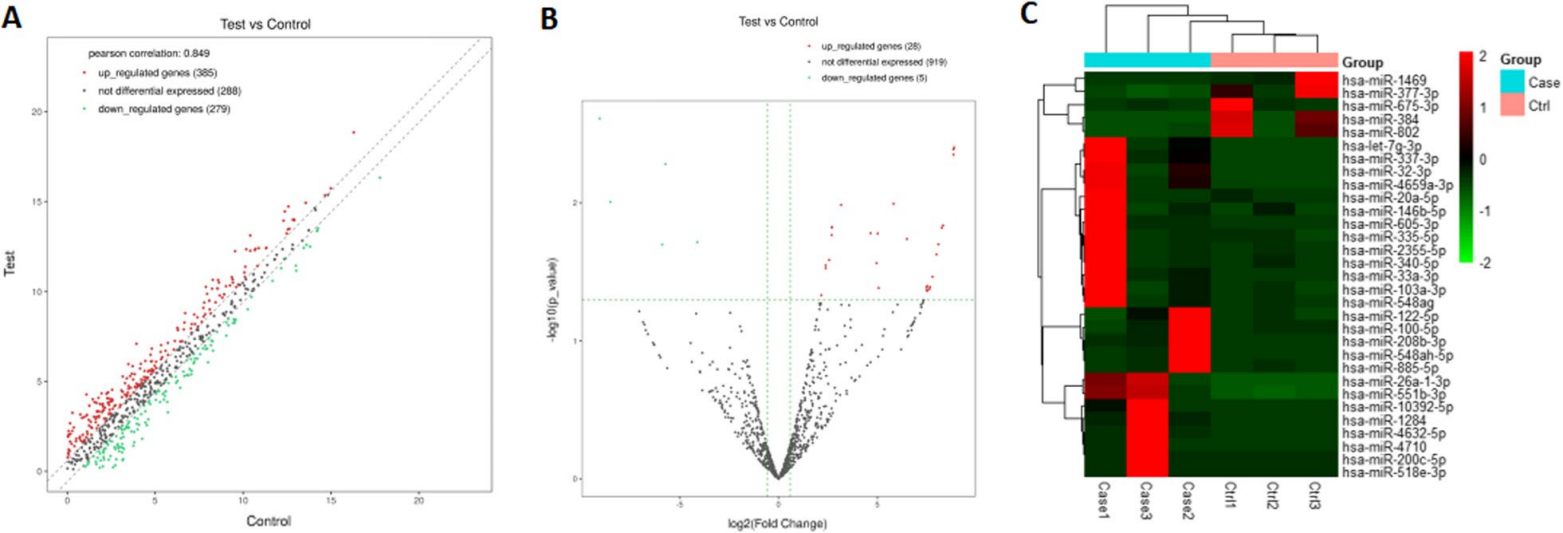
Differentially expressed miRNAs in FHCM patients. **a** Scatter plots of differentially expressed miRNAs in FHCM and control groups. Green dots represent the significantly downregulated miRNAs, red dots represent the significantly upregulated miRNAs, and gray dots represent the non-significantly expressed miRNAs. Two oblique dashed lines divide up and down (2 times) and unchanged miRNAs. **b** Volcano plot of differentially expressed miRNAs in FHCM and control groups. The two vertical green lines represent upregulated (right) and downregulated (left) miRNAs, respectively, and the green parallel lines correspond to the *p* value. Green dots represent the significantly downregulated miRNAs, red dots represent the significantly upregulated miRNAs, and gray dots represent the non-significantly expressed miRNAs. **c** Hierarchical clustering plot shows that the significant differentially expressed miRNAs. (Test for FHCM patients, and Control for healthy group). Each column represents the expression profile of a tissue sample, and each row corresponds to a miRNA. Red means high expression level and green means lower levels

A total of 952 miRNAs were detected in our miRNA sequencing analysis of peripheral blood plasma samples from the three FHCM patients with *MYBPC3* gene mutations and the three age-and sex-matched healthy controls. Of these, 385 miRNAs were upregulated, 279 were downregulated, and 288 were unchanged in the FHCM patients compared to the control subjects with fold change > 1.5 (Fig. 2a).

### Differentially expressed miRNAs

EdgeR was used to analyze the differential miRNA expression between groups. The threshold for screening significantly differentially expressed miRNAs was set at *p* < 0.05, with a FC > 1.5. A total of 919 non-significantly differentially expressed miRNAs and 33 significantly differentially expressed miRNAs (28 upregulated and 5 downregulated) were identified in FHCM patients (Fig. 2b). The hierarchical clustering plot shows that the significant differentially expressed miRNAs (Fig. 2c).

### RT-qPCR validation of differentially expressed miRNAs

**Fig. 3:**
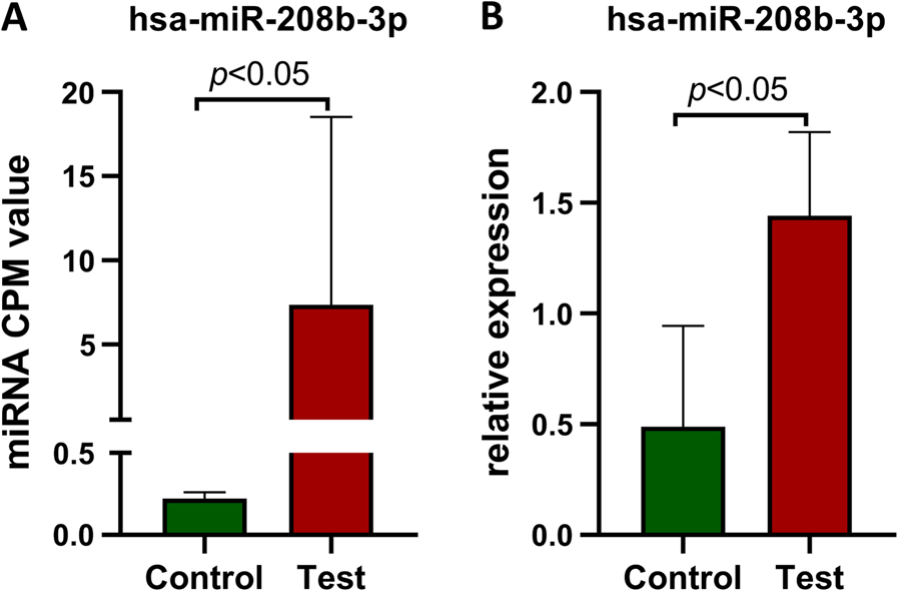
Hsa-miR-208b-3p is upregulated in FHCM patients plasma. **a** The CPM values of has_miR-208b-3p in miRNA-seq analysis data. Green bar chart indicates the mean value of healthy group CPM value and red indicates FHCM group. **b** RT-qPCR validation result suggests hsa-miR-208b-3p is upregulated in the peripheral blood plasma of FHCM patients with MYBPC3 gene mutations

According to previous reports and screening conditions (*p* < 0.05 and FC > 1.5) [23], we identified and used RT-qPCR to validate the expression of a total of 10 significantly differentially expressed miRNAs (miR-103a-3p, miR-122-5p, let-7g-3p, miR-26a-1-3p, miR-675-3p, miR-200c-5p, miR-208b-3p, miR-2355-5p, miR-146b-5p, and miR-377-3p) (Fig. 3a). The expression trends of let-7g-3p, miR-103a-3p, miR-122-5p, miR-146b-5p, miR-26a-1-3p, miR-208b-3p, hsa-miR-675-3p, and hsa-miR-377-3p were consistent with the miRNA-seq analysis, but only miR-208b-3p was significantly different (*p* < 0.05) between the FHCM and control groups. The expression trends of miR-2355-5p and miR-200c-5p obtained by RT-qPCR were not consistent with the miRNA-seq analysis (Fig. 3b).

### Prediction of target genes and signaling pathways of miR-208b-3p

**Fig. 4:**
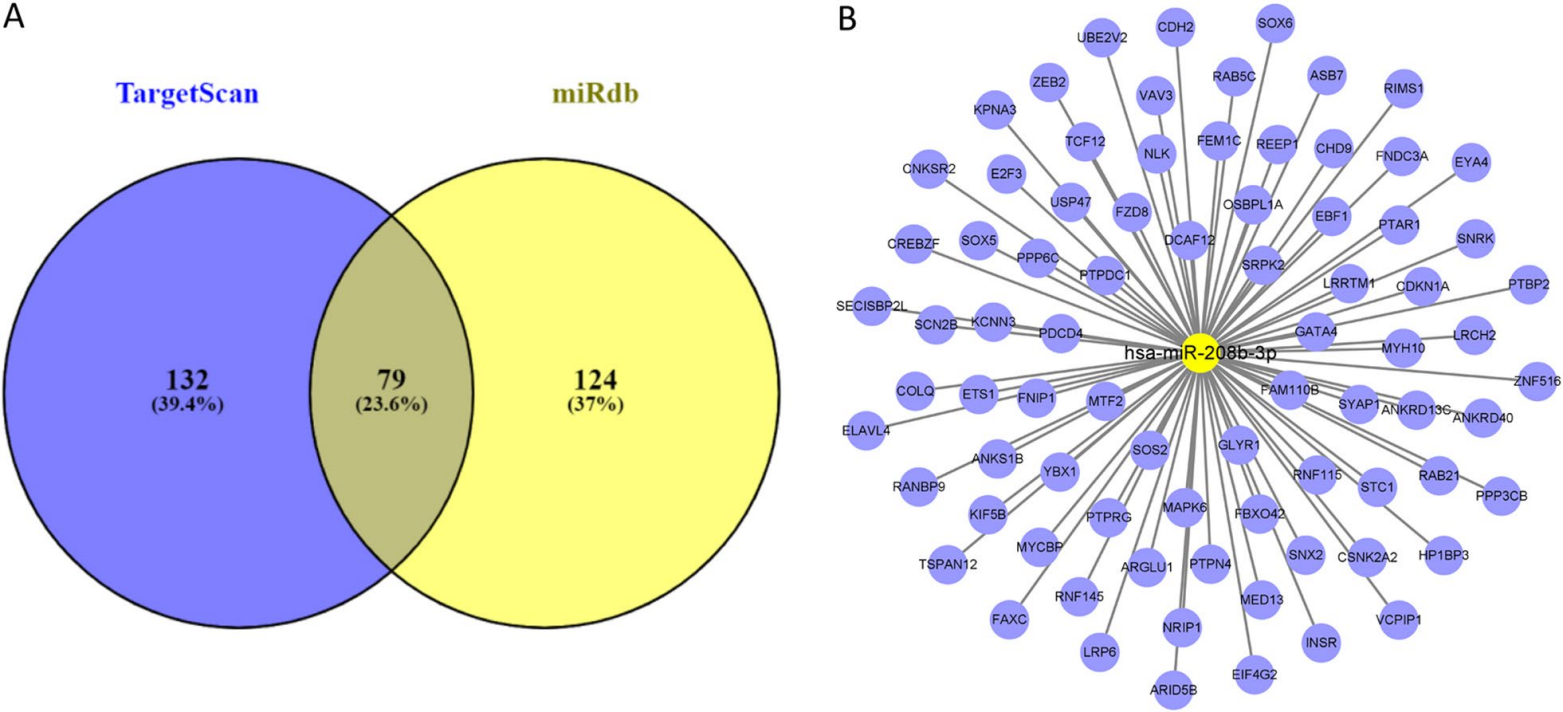
Hsa-miR-208b-3p and its target genes network. **a** Venn plot show the intersection result of miR-208b-3p target genes predicted from Targetscan and miRdb. **b** miR-208b-3p and 79 target gene network. Yellow circle is hsa-miR-208b-3p, and blue circles is the targeted genes predicted by Targetscan and miRdb database

Target genes of miR-208b-3p were predicted using the TargetScan and miRDB databases, filtered with TargetScan contex++ score <− 0.05 and miRDB score > 50, and the resulting target genes were intersected (Fig. 4a). After removing duplicate genes, a total of 79 target genes were finally obtained (Fig. 4b).

**Fig. 5:**
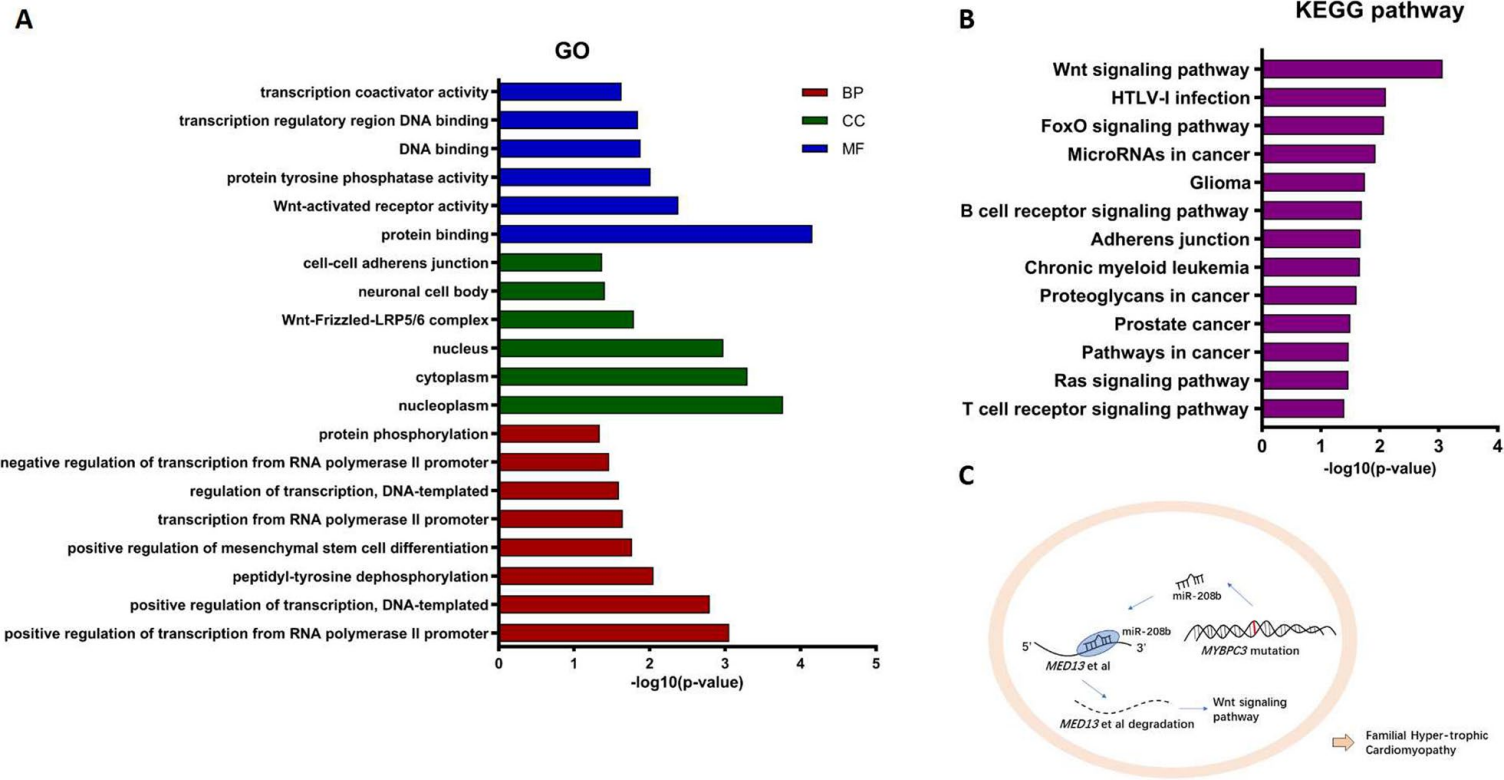
Functional enrichment analysis result indicates the complex regulatory functions of miR-208b-3p in the MYBPC3 mutations FHCM patients. **a** GO analysis of miR-208b-3p. The red bars represent biological process terms, the green bars represent cellular components terms, and the blue bars represent molecular function terms. Horizontal axis means enrichment score (equal to − log_10_(*p* value)). Every bar means every enriched GO terms. **b** KEGG pathway analysis of miR-208b-3p. Horizontal axis means enrichment score (equal to − log_10_(*p* value)). Each bar indicates one pathway terms. **c** The schematic illustration of miR-208b-3p

As shown in Fig. 5a, GO analysis of the predicted target genes showed that the target factors of miR-208b-3p were mainly involved in the regulation of transcription from RNA polymerase II promoter, regulation of transcription, protein phosphorylation, et al., processes, mainly localized in nucleoplasm. And their molecular functions were mainly related to protein binding and interactions. KEGG pathway analysis showed that miR-208B-3p was primarily enriched in the Wnt and FoxO signaling pathways, as well as HTLV-1 infection (Fig. 5b), and we added a schematic illustration in Fig. 5c.

## Discussion

HCM is a hereditary cardiomyopathy characterized by ventricular asymmetric hypertrophy, with mutations in the *MYBPC3* gene detected in 40% of all FHCM cases [24]. Previous studies have shown that, in most cases, HCM with *MYBPC3* gene mutations is a late onset disorder with mild myocardial hypertrophy, a lower rate of sudden cardiac death, and relatively benign disease progression [25, 26]. However, in the present study, echocardiography of the three probands showed that the degree of myocardial hypertrophy was severe (the thickest part of the ventricular wall was > 30 mm) and all were obstructive, and their troponin I increased and had a certain degree of myocardial injury. Among these probands, two families had a history of sudden death. In contrast to other reports [27, 28], our findings indicate that the clinical phenotypes of FHCM with *MYBPC3* gene mutations in this study were severe, suggesting that the clinical phenotypes of FHCM in patients with the same gene mutation are variable, and may be affected by many factors including environmental cues, genetic modifiers, and protein post-transcriptional modification.

Previous studies have shown that miRNAs are abundant in the heart and play important roles in a variety of cellular events, such as cell proliferation, apoptosis, autophagy, and cell metabolism. In this study, a total of 952 miRNAs were detected in a miRNA-seq analysis of three cases of FHCM with *MYBPC3* gene mutations. Of these 952 miRNAs, 33 differentially expressed miRNAs were identified in the FHCM group, 10 of which were chosen for RT-qPCR validation. We found that miR-208b-3p was the only statistically significant upregulated miRNA, which was consistent with the results of the miRNA-seq analysis. These results suggest that miR-208b-3p expression is upregulated in the peripheral blood of FHCM patients with *MYBPC3* gene mutations. Thus, we speculated that miR-208b-3p might be a regulator of cardiac hypertrophy. The miR-208 family is a member of a cardiac-specific group of miRNAs that includes miR-208a and miR-208b. MiR-208a is encoded by an intron of the alpha-cardiac muscle myosin heavy chain gene (*Myh6*), while miR-208b is encoded by an intron of the beta-cardiac muscle myosin heavy chain gene (*Myh7*). MiR-208a and miR-208b are expressed in both the left atrium and left ventricle [29], and they share a similar sequence that allows them both to regulate myocardial hypertrophy by targeting Thrap1 and myostatin [30]. In addition, overexpression of miR-208a is sufficient to induce myocardial remodeling by regulating the expression of hypertrophic pathway components [31], indicating that miR-208a plays a pathogenic role in the development of cardiac hypertrophy.

Through bioinformatics analysis, we predicted the target genes of miR-208b-3p and found that miR-208b-3p was mainly enriched in 79 target genes, including *UBE2V2*, *MED13*, *YBX1*, *CNKSR2*, *GATA4*, and *SOX5/6, *et al. GO analysis revealed that the functions of these 79 target genes involve regulation of regulation of transcription from RNA polymerase II promoter, regulation of transcription, and localized in nucleoplasm and cytoplasm; their molecular functions were mainly related to protein binding and Wnt-activated receptor activity, et al. These predictions illustrate the complex regulatory functions of differentially expressed miRNAs in the pathogenesis of FHCM with *MYBPC3* gene mutations. KEGG pathway analysis revealed that miR-208b-3p was mainly enriched in 13 pathways, including the Wnt and FoxO signal pathways, as well as HTLV-1 infection, et al. The Wnt signaling pathway includes the canonical Wnt/β-catenin pathway and non-canonical pathways (including the Wnt/calcium pathway and Wnt/planar cell polarity pathway) and is a highly conserved signal transduction pathway. Zhang et al*.* [32] reported that the Wnt/calcium pathway activates myosin enhancer factor 2 (MEF-2), which plays an important role in cardiac hypertrophy. Wnt5a, another component of Wnt signaling pathway, also induces cardiac hypertrophy by activating the Wnt/PCP pathway [33]. Dawson et al*.* [34] showed that conditional deletion of beta-catenin inhibited the Wnt/beta-catenin pathway and attenuated hypertrophic responses. The study has showed that over expresssion of miR-208b-3p increased the expression levels of Wnt/beta-catenin signaling pathway-related proteins (Wnt3a, Wnt5a and beta-catenin) by suppressing the expression of mediator complex subunit 13(MED13) [35]. Therefore, our findings suggest that miR-208b-3p promotes myocardial hypertrophy by suppressing the activity of its target genes, such as *MED13*, to activate the Wnt signaling pathway (Fig. 5c).

### Limitations

Some limitations of this study should be noted. First, we performed this study with a limited number of patients. Second, we did not study other gene and non-gene mutations associated with FHCM. Therefore, future studies with a larger sample size are required to further investigate the possible associations of miRNAs with FHCM linked to different gene and non-gene mutations. Third, we did not perform any functional assays of miRNA target genes identified in this study. In the study, we found that miRNA-208b-3p is significantly upregulated in FHCM patients. miR-208b is known as a biomarker of myocardial injury, therefore the role of miR-208b should be investigated further.

## Conclusion

In the present study, we identified differentially expressed miRNAs in FHCM *MYBPC3* gene mutations, and found that miRNA-208b-3p is significantly upregulated in FHCM patients. We further uncovered that miRNA-208b-3p potentially activates the Wnt signaling pathway through suppressing the activity of its target genes to promote cardiac hypertrophy.

## Data Availability

The datasets used and/or analyzed in the present study are available from the corresponding author on reasonable request.

## Abbreviations

FHCM: Familial hypertrophic cardiomyopathy
miRNA: MicroRNA
MYBPC3: Myosin-binding protein C3
qPCR: Real-time quantitative PCR detecting system
GO: Gene ontology
KEGG: Kyoto Encyclopedia of Genes and Genomes
TNNT2: Cardiac troponin T
TNNI3: Cardiac troponin I
TPM1α: Tropomyosin
ACTC: Cardiacα-actin
MYL: Myosin regulatory light chain
CMR: Cardiac magnetic resonance
LVH: Left ventricle hypertrophy
FC: Fold change
